# Effect of the Hydration Shell on the Carbonyl Vibration in the Ala-Leu-Ala-Leu Peptide

**DOI:** 10.3390/molecules26082148

**Published:** 2021-04-08

**Authors:** Irtaza Hassan, Federica Ferraro, Petra Imhof

**Affiliations:** 1 Institute for Theoretical Physics, Freie Universtiät Berlin, Arnimallee 14, 14195 Berlin, Germany; irtaza06@zedat.fu-berlin.de; 2 Computer Chemistry Center, Friedrich-Alexander University (FAU) Erlangen-Nürnberg, Nägelsbachstrasse 25, 91052 Erlangen, Germany; federica.ferraro@fau.de

**Keywords:** infrared spectra, amide band, hydrogen bonds

## Abstract

The vibrational spectrum of the Ala-Leu-Ala-Leu peptide in solution, computed from first-principles simulations, shows a prominent band in the amide I region that is assigned to stretching of carbonyl groups. Close inspection reveals combined but slightly different contributions by the three carbonyl groups of the peptide. The shift in their exact vibrational signature is in agreement with the different probabilities of these groups to form hydrogen bonds with the solvent. The central carbonyl group has a hydrogen bond probability intermediate to the other two groups due to interchanges between different hydrogen-bonded states. Analysis of the interaction energies of individual water molecules with that group shows that shifts in its frequency are directly related to the interactions with the water molecules in the first hydration shell. The interaction strength is well correlated with the hydrogen bond distance and hydrogen bond angle, though there is no perfect match, allowing geometrical criteria for hydrogen bonds to be used as long as the sampling is sufficient to consider averages. The hydrogen bond state of a carbonyl group can therefore serve as an indicator of the solvent’s effect on the vibrational frequency.

## 1. Introduction

Peptides are often used as small, tractable model systems for proteins in order to study their conformational dynamics and the dynamics of the surrounding water molecules, which play a key role in protein function [1]. Typically, protein or peptide dynamics take place over longer timescales, whereas the timescales of water dynamics are around picoseconds, mainly due to high mobility of water molecules and frequent changes in the hydrogen bonding state [2,3,4]. To probe the conformational state of a peptide, infrared (IR) spectroscopy of the so-called amide region is employed. The vibrational fingerprints of this region are due to the motions of the groups involved in the peptide bond, that is, carbonyl, C=O, and N−H group stretching and bending motions. Depending on the backbone conformation of a peptide, e.g., a fully formed α-helix or a β-sheet, the characteristic frequencies of the bands in this region differ, in principle, allowing an assignment of the observed conformations. For larger and flexible peptides, there are, however, many possible conformations conceivable, and it is not a priori clear which one dominates and whether and how these interchange. While time-resolved IR spectroscopy techniques provide a time resolution that allows us to measure structural dynamics of proteins and peptides in a solvated environment on picoseconds timescales [5], the assignment of the timescales to the underlying processes, and even more simply, the probable conformations, call for an accompanying approach.

Molecular dynamics (MD) simulations at the atomic level are enjoying great popularity in evaluating conformational dynamics of peptides and when using empirical force fields and sophisticated analysis methods, such as Markov state models (MSMs), molecular simulations have proved successful in this task [6,7,8]. In order to simulate (and assign) infrared spectra, the usefulness of empirical force-field based simulations is, however, limited, since they cannot account reliably for the changes in electron density and the corresponding infrared intensity associated to a molecular vibration.

To this end, first-principles MD simulations provide a platform to accurately analyze the dynamics at atomic level and moderate timescale and allow for the computation of IR spectra of small systems in explicit solvent [9,10,11]. These simulations include explicit solvent, finite temperature, and anharmonic effects that are otherwise missing in a normal modes analysis (possibly carried out with an even more accurate potential, though) [12]. Usually, a maximally localized Wannier functions scheme is used to estimate the instantaneous molecular dipole vectors [13]. The calculation of IR spectra using the trajectory of molecular dipole vectors with the help of a Fourier transform is straightforward [12]. This method yields not only nicely resolved frequency bands, if the underlying trajectory is sufficiently long, but also a dynamic average over this trajectory.

For the assignment of different conformations of a peptide, one therefore needs to rely on a set of first-principles simulations, in which each conformation, obtained, e.g., from sampling with empirical force fields, is simulated separately [14]. Such an approach is feasible if the time scale of the conformational dynamics is beyond that of the simulation time the spectra calculation is based on, and all other processes that may effect the vibrational signature are fast enough to average out. The first is usually the case for the backbone dynamics of peptides, but not necessarily for the fluctuations in the side chains. Furthermore, the dynamics of the surrounding solvent, water, is on a timescale that is about feasible in first principle simulations but may be impacted by the interaction with the solute.

Likewise, the vibrational frequencies of polar groups such as C=O, N−H, charged termini, etc., are sensitive to their interaction with the surrounding water, classified by, e.g., their hydrogen bonding states [5,15]. The fluctuations in molecular motions of solvent molecules give rise to fluctuations in the vibrational frequencies of polar groups. Similarly, the vibrational frequencies of individual polar groups are influenced by the presence of the surrounding polar groups, either due to direct or indirect vibrational couplings [16], or water-mediated intramolecular interactions [17]. The amide I vibration is depicted by a prominent band in the IR spectra, and is governed by the motion of carbonyl groups. This band is also sensitive to the hydrogen bonding state of the peptide, and due to its intensity in the IR spectrum, a popular marker for the peptide’s conformation. It is therefore of interest to study variations in the characteristic amide I frequency, due to changing interactions with the solvent.

In order to obtain both time and frequency information, an analysis of the instantaneous frequencies is required. The localization of the frequency of an input signal in time can be achieved by another integral transform approach called wavelet transform [18,19,20] that has recently gained popularity in the molecular dynamics community [21,22,23,24,25,26].

Many experimental and computational efforts have been made to better understand the solute–solvent interaction and the consequences on the amide I region, mainly on short peptides such as N-methyl amide (NMA), di- or trialanine [27,28] and other small model peptides [29,30,31,32]. Several approaches have been used to quantitatively determine how the hydration induced shift on the amide I vibrational band is related to the intermolecular interactions between solute and solvent. Such interactions can easily be computed between individual molecules, but, unless for empirical potentials, a dissection of interaction with groups of atoms within one molecule is more involved. To this end, energy decomposition schemes based on quantum mechanical calculations and linear scaling techniques to take into account electrostatics, polarization, and charge transfer terms have been successfully applied to NMA [33]. An alternative way is to implement a molecular fragmentation method and calculate these interactions using quantum mechanical methods. In the approach used in our study, we take out a small fragment of the full molecule to describe the impact of intermolecular interactions on the amide I mode. In another study [34], a computational protocol (ONIOM) aimed at the quantitative reproduction of the spectra of bio-organic and hybrid organic/inorganic molecular systems with a proper account of the variety of intra- and intermolecular interactions was applied. By static density functional theory calculations of NMA and NMA—water complexes, the impact of hydrogen bonding on the C=O and N−H as well as the amide bond geometry and on the amide I, amide II, and amide III vibrations has been studied [35].

In this work, we investigate the vibrational signature of the small peptide Alanine-Leucine-Alanine-Leucine (ALAL) and the effect of the fluctuations of solute molecules and hydrogen bonding states on the amide I frequencies by employing a combination of first-principles MD simulations, fragmentation methods to quantify interaction energies, and geometrical analyses.

## 2. Materials and Methods

### 2.1. Molecular Mechanics Simulations

We performed classical MD simulations of the Ala-Leu-Ala-Leu (ALAL) peptide in a cubic simulation box of explicit water (1477 molecules modeled as TIP3P [36] water) employing the AMBER 99SB-ILDN [37,38] force field. We used a minimum distance of 1 nm between the solute and the box’s periodic boundaries, resulting in side length of 3.61 nm and a total number of atoms of 4492. Water hydrogen atoms and polar hydrogen atoms of the peptide ($ND_{3},N-D$) were modeled with deuterium mass. For Lennard-Jones interactions and electrostatic interactions (Particle-Mesh Ewald [39,40] with a grid spacing of 0.16 an interpolation order of 4), we used a cutoff value of 1 nm. The system was minimized and equilibrated for 500 ps. We ran six 2.5μs-long MD simulations, which result in a total simulation time of 15μs. A V-rescale [41] thermostat was applied to control the temperature at 300 K (NVT ensemble). The positions of the solute atoms were saved to file every 0.25 ps. No constraints were applied, and the leap-frog integrator with a time step of 1 fs was employed using the GROMACS simulation package [42].

Free energy distributions are calculated as

$$F=-k_{b}T\ln Z$$

from the two-dimensional histogram of the ψ and ϕ angles, where $k_{B}$ is Boltzmann’s constant, *T* is the temperature and $Z=\frac{H_{r}}{H_{0}}$ is the count in the histogram, relative to the state with maximal counts.

### 2.2. First-Principles Molecular Dynamics Simulations

The first-principle MD simulations were performed using the CP2K package [43,44]. We employed the default Gaussian and plane waves (GPW) electronic structure method [45] as implemented in the Quickstep module [46]. We used a double zeta valence plus (DZVP) basis set, and interaction between valence and core electrons is described by Geodecker–Teter–Hutter (GTH) norm-conserving pseudopotentials. A plane wave expansion for the charge density is employed using an energy cutoff of 500 Ry. BLYP with Grimme’s D3 dispersion correction is used as exchange–correlation functional [47,48,49]. It provides a robust electronic representation for dynamical spectroscopy of hydrated peptides [50,51]. During the NVT equilibration, the temperature was controlled to be at 300 K using a chain of three Nosé–Hoover thermostats [52] with a time constant for the thermostat chain of 100 fs. CP2K default values of other thermostat parameters are used. To keep the computational cost of the first-principles simulations moderate, the box size and the number of water molecules are smaller than in the classical simulations but still large enough to avoid interactions of the periodic images. The cubic simulation box had a minimum distance of 0.4 nm between the solute and the box’s boundaries, and periodic boundary conditions were applied in all three dimensions. This results in cubic box of side lengths 22.2Å and 328 solvent molecules. Albeit using a small minimum distance between the solute and the box’s boundaries, the total number of atoms jumps to 1045. For such a large number of atoms, the computational cost to perform first-principles MD simulations is already high. Like in the classical MD simulations, water hydrogen atoms and polar hydrogen atoms of the peptide ($ND_{3},N-D$) were modeled with deuterium mass.

First, the system was energy minimized using a conjugate-gradient algorithm where the positions of the ALAL atoms were fixed. This allows the solvent molecules to relax around the peptide and find energy favorable positions. We performed a 5 ps NVT equilibration run from the minimized system, during which the solute was kept fixed to avoid the transition to an undesired conformation, followed by the production run of 50 ps in an NVE ensemble. The time-step for the numerical integration was 0.5 fs. Atom positions were saved every step, and every fifth step Wannier localization was performed to monitor the changes in the dipole moment [53,54,55,56,57]. The gathered Wannier centers and position data sets are large enough to compute the reliable IR and power spectra of the amide region of ALAL in explicit solvent. Notably, the hydrogen bond breaking and water rearrangement, which occurs at a 2–3 ps timescale [50], is adequately sampled to have a prominent effect on the resulting spectra. In TRAVIS, these saving rates together with the correlation resolution of 1024 and 4096, allow a spectral resolution of infrared and power spectra of ∼1.63 cm${}^{-1}$ and ∼2.04 cm${}^{-1}$, respectively.

#### Constrained Simulations

We performed three constrained simulations, launched from a snapshot from the previous, unconstrained trajectory. The overall atomic position of either a single or two water molecules was fixed.

(1) One $D_{2}O$ molecule constrained to a distance of 3.0Å from the $C_{2}=O_{2}$ group and the acceptor–donor hydrogen angle, ∠ADH, constrained to $∼30^{0}$.

(2) One $D_{2}O$ molecule constrained to a distance of 3.2Å from the $C_{2}=O_{2}$ group and the acceptor–donor hydrogen angle, ∠ADH, constrained to $∼0^{0}$ and another water molecule, only distance constrained at 4.0Å from the $C_{2}=O_{2}$ group so as to prevent another water molecule to enter the hydration sphere and to construct a single hydrogen bond situation.

(3) Two $D_{2}O$ molecules constrained to a distance of 2.6Å from the $C_{2}=O_{2}$ group and the acceptor–donor hydrogen angle, ∠ADH, constrained to $∼0^{0}$.

We performed another 5 ps NVT equilibration run for each constraint scenario, followed by the production run of 20 ps each in an NVE ensemble. For these constrained simulations, no Wannier localization was performed.

For both, unconstrained and constrained simulation, Fourier-transform-based spectra and the structural analysis were conducted using the TRAVIS program [58,59] and our own TCL, Python, and Java scripts.

### 2.3. Wavelet Analysis

For the calculation of spectrogram and instantaneous stretching frequencies of carbonyl groups, we used wavelet theory. For any signal in time domain f(t), it can be described as:

(1) $$W\left(\tau ,s\right)=\frac{1}{\sqrt{\left|s\right|}}\int_{-\infty }^{+\infty }dtf\left(t\right)\overset{*}{\psi }\left(\frac{t-\tau }{s}\right)$$

ψ(t) is the so-called mother wavelet, which is translated and compressed/dilated by the τ and *s* parameter, respectively. The τ parameter localizes the frequency in time, and 1/s is proportional to frequency. We used the Morlet–Gabor mother wavelet [19], which has been successfully applied in many previous studies to calculate instantaneous stretching frequencies. It is defined as

(2) $$\psi \left(t\right)=\pi^{-\frac{1}{4}}e^{i\omega_{0}t}e^{-t^{2}/2\sigma^{2}}$$

where $\omega_{0}$ represents the main oscillation frequency of the plane wave, and σ represents the width at half-height of the Gaussian time window.

For a mother wavelet to be applicable for wavelet analysis, it should be localized (i.e., have finite energy) and admissible (i.e., have zero mean). In the above-described wavelet, parameter σ controls its locality properties, and it also directly affects the time–frequency resolution of a spectrogram.

The discretized version of the continuous WT is given by:

(3) $$W\left(n,s\right)=\sum_{n^{\prime }=0}^{N-1}f\left(n^{\prime }\cdot \delta t\right)\overset{*}{\psi }\left[\frac{(n^{\prime }-n)\cdot \delta t}{s}\right]$$

The product $n^{\prime }\delta t$ shows the total time at the $n^{\prime }th$ time step, which localizes the signal in time, and consequently, WT gives the frequency content of a signal over a Gaussian time window centered at nδt.

The wavelength for the Morlet–Gabor set of basis functions is defined as:

(4) $$\lambda =\frac{s4\pi }{\omega_{0}+\sqrt{2+\omega_{0}^{2}}}$$

We used σ=8, and $\omega_{0}=2\pi$ which yields the value of corresponding effective frequency ν=1.01/s. For the detailed theory and implementation of wavelet analysis, see [20]. The value of *s* is found such ‘that [it] maximizes the modulus ${\left|W\left(n,s\right)\right|}^{2}$ of the wavelet at a given time step n’. The corresponding value of 1/s is taken as the “instantaneous stretching frequency” [24]. To calculate the spectrogram and instantaneous frequencies using the wavelet transform, we used the FORTRAN code developed by the group of Pagliai [24].

### 2.4. Normal Modes

For ALAL bound to different numbers of water molecules, corresponding to topologies of hydrated ALAL observed in the first-principle MD simulations, we carried out a normal mode analysis using the Gaussian program package [60]. The snapshots were optimized (convergence criterion $3.00E-04E_{H}/Å$) in implicit water (modeled by a polarisable continuum model, PCM, with a dielectric of ε=80) at the DFT level of theory. On the optimized geometries, a frequency calculation was performed in which all polar hydrogen atoms are assigned an atomic mass of 2. Like for the first-principles simulations, the BLYP exchange–correlation density functional was used. To also use a basis set of comparable double-zeta quality, a cc-pVDZ basis set was employed for all these static calculations.

### 2.5. Interaction Energies

**Figure 1:**
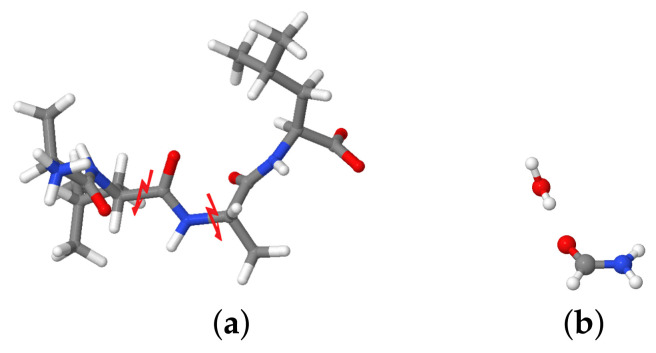
Fragmentation of the ALAL peptide for the calculation of interaction energies with individual water molecules. (**a**) Positions for splitting the fragment (**b**) Resulting H-saturated −CONH group with one water molecule.

The molecular fragmentation method was employed to calculate the interaction energies of the central carbonyl group with the closest water molecules for different snapshots of the system, taken at 50 fs intervals. In this study, we consider as fragment of the molecule the −CONH group to preserve the electronic structure of the peptide bond. First, the molecule was fragmented and hydrogen caps were inserted at the broken C–C and N–C bonds to preserve the valency of the fragment. Then, the water molecule of interest was added to the fragment according to its distance from the C=O group (see Figure 1). A similar approach was used in [61]. Our fragmentation approach assumes that the other polar groups are far enough from the fragment to not affect the interaction energies with the water molecules. Interaction energies were calculated with Gaussian 16 [60] for comparability at the same level as the normal modes, that is, BLYP/cc-pVDZ level of theory.

### 2.6. Hydrogen Bonds and Water Topology

For the analysis of hydrogen bonds, we used a geometric criterion of 2.5Å as the maximal distance between the hydrogen atom and the acceptor and a donor–hydrogen–acceptor angle of $45^{\circ }$ as the maximal deviation from linearity. As is discussed in this paper, geometric criteria are a somewhat arbitrary choice, but allow for fast and efficient evaluation of hydrogen-bonding states. The chosen criteria are common in simulations of peptides and proteins in solution and turn out to be justified by the calculated interaction energies (see results).

A hydrogen-bonded state of a polar group of the ALAL peptide is defined by the number of water molecules that are simultaneously hydrogen-bonded to that group. These water molecules constitute the “first solvation shell” around that polar group.

Water bridges between polar groups of the ALAL peptide were determined using a module implemented in MDAnalysis [62]. This code uses an approach similar to breadth-first search, where the first hydrogen-bonded water to the $C_{2}=O_{2}$ group is selected (selection 1), followed determination of the second solvation shell as well as any other hydrogen bonding partners from $C_{2}=O_{2}$ group (selection 2). After that, the next solvation shell, as well as water molecules hydrogen-bonded to selection 2, are detected. This process is repeated until the desired order of water bridges (here up to seven) is reached [62].

All other analyses, except where indicated, were performed using our own Java code using JgraphT [63] and in-house Python code. Plots were generated using matplotlib [64].

## 3. Results

### 3.1. Conformational Analysis

**Figure 2:**
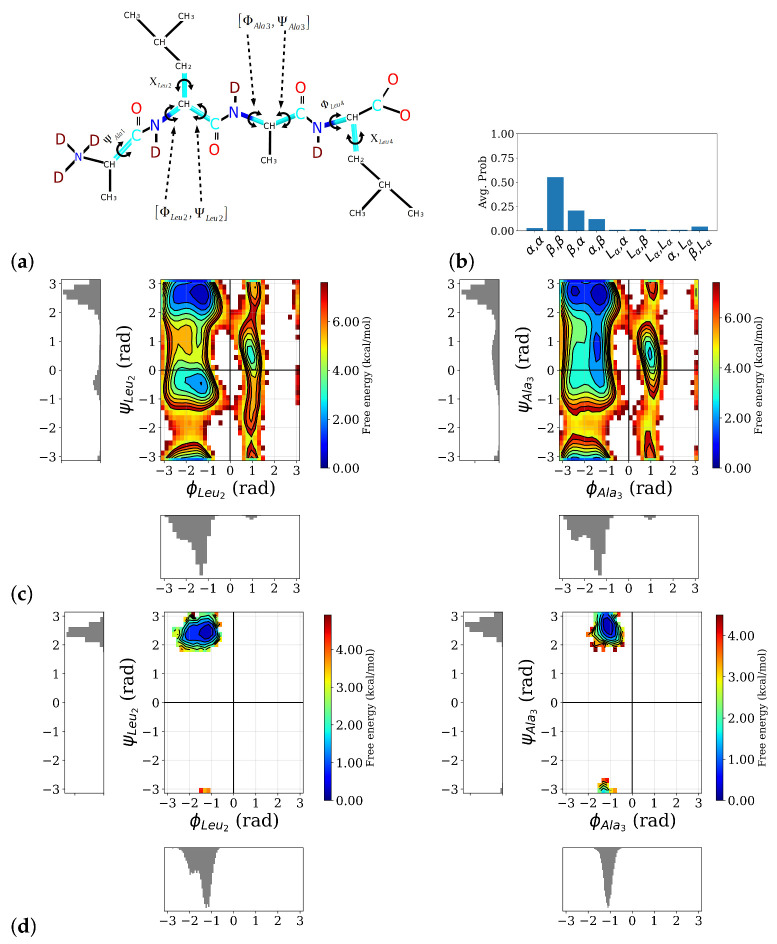
Distribution of backbone conformation of the ALAL peptide. (**a**) Definition of backbone, ψ,ϕ and side chain, χ, torsional angles, (**b**) probability distribution of different backbone conformations as observed in the classical MD simulations, the first label refers to the first peptide bond, i.e., the $\psi_{Leu2},\phi_{Leu2}$-pair, and the second one to the second peptide bond, i.e., the $\psi_{Ala3},\phi_{Ala3}$-pair, (**c**) free energy profile and marginal probability distributions of the central ψ,ϕ torsion angles (for the other torsion angles see Figure S1), (**d**) two-dimensional free energy distributions of the two ψ,ϕ-pairs, computed from the first-principles simulations, confirming the peptide stays in the β,β conformation.

Analysis of the MD simulations of the ALAL peptide in water with an empirical force field shows that the conformational space of the backbone torsion angles ψ,ϕ (see Figure 2a) for definition) is well sampled and all sterically “allowed” regions in a Ramachandran plot, i.e., around the angles that define α-helix (α: $\phi \approx -57^{\circ };\psi \approx -47^{\circ }$), β-sheet (β: $\phi \approx -130{}^{\circ };\psi \approx +140$), or left-handed helix (*L*:$\phi \approx 80{}^{\circ };\psi \approx +70$) conformations, were visited and regions corresponding to secondary structures such as α-helix, β-sheet, or left-handed helix, show the lowest free energies (see Figure 2c). The side chain torsion angles, however, exhibit only significantly populated state in the first-principles simulations, whereas in the longer classical simulations, two well-popluated states can be observed for for $\chi_{Leu2}$ and $\chi_{Leu4}$ (see Figure S1). Among the possible backbone conformations, a conformation in which the first and second peptide bonds are in a β-sheet-like conformation, labeled as β,β, clearly dominates (see Figure 2b). The second and third most probable conformations, β,α, and α,β, respectively, both also have one of the two peptide bonds in a β-conformation, indicating a preference for a more “stretched” conformation in this ALAL peptide, likely due to the steric demands of the bulky Leu side chains.

This conformation is preserved in the course of the first-principles simulations, launched from the β,β conformation as can be seen in the two-dimensional free energy distributions of the two ψ,ϕ-pairs (see Figure 2d). Because of its predominance, we confine our spectroscopic analysis to the β,β conformation and from now on drop the label β,β.

### 3.2. Vibrational Analysis

**Figure 3:**
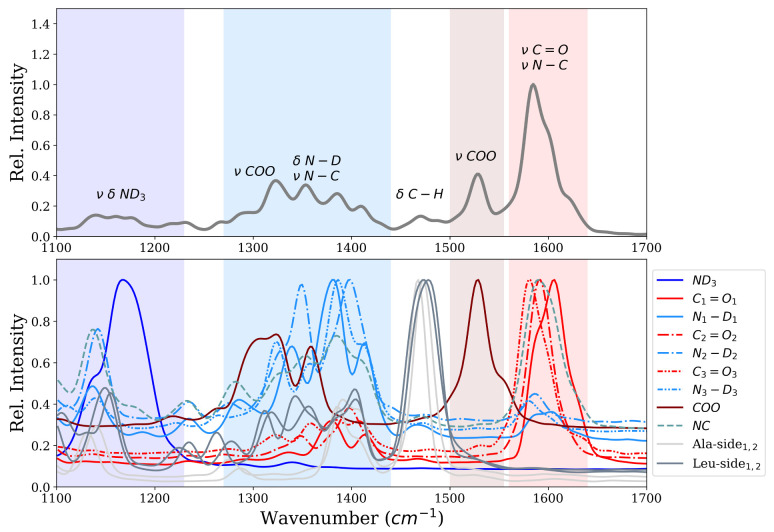
Infrared spectrum (**top**) and power spectrum (**bottom**) of the ALAL peptide in water in β,β conformation.

Figure 3 shows the infrared spectrum computed from first-principle MD simulations of the ALAL peptide in deuterated water. The amide I region shows one intense band centered at ∼1600 cm${}^{-1}$, which can be attributed to the carbonyl stretch vibration (νC=O), and another one at ∼1528 cm${}^{-1}$ which we assign to the stretch vibration of the carboxyl group (νCOO). Also bands in the amide II and amide III region between ∼1250–1440 cm${}^{-1}$ and ∼1100–1250 cm${}^{-1}$, respectively, are visible. According to the computed power spectrum, these bands correspond to vibrations of the N−D/N−C groups and the N-terminal $ND_{3}$ group, respectively. There is also a significant contribution of the carboxyl group to the bands of the amide II region, as can be seen from the power spectrum (Figure 3 bottom panel). The motion of both the Ala and the Leu side chain have a large peak at ∼1470 cm${}^{-1}$ in the power spectrum which, due to the low change in dipole moment of these unpolar groups, translates to only little intensity in the infrared spectrum.

Closer inspection of the motions, i.e., by means of power spectrum, responsible for the most prominent band at ∼1600 cm${}^{-1}$, the “carbonyl band”, reveals this band to be a superposition of the motion of the three carbonyl groups and a component along the peptide N−C bond. Note, however, that the same C−atom is part of the N−C and the C=O vibration. The frequencies of the three carbonyl groups $C_{1}=O_{1}$, $C_{2}=O_{2}$, $C_{3}=O_{3}$ are ∼1606 cm${}^{-1}$, ∼1592 cm${}^{-1}$ and ∼1580 cm${}^{-1}$ respectively. The three C=O groups in the ALAL peptide hence move with frequencies that differ by ∼12–14 cm, not enough to be resolved in the C=O band of the IR spectrum, but sufficiently large to wonder why this is the case.

A normal mode calculation of the ALAL peptide in the β-conformation in implicit solvent (see Figure S2) shows the frequencies of the three carbonyl groups $C_{1}=O_{1}$, $C_{2}=O_{2}$, $C_{3}=O_{3}$ are ∼1645 cm${}^{-1}$, ∼1631 cm${}^{-1}$, and ∼1619 cm${}^{-1}$, respectively. Note that the $C_{2}=O_{2}$ group also contributes to the normal mode at ∼1619 cm${}^{-1}$ and the $C_{3}=O_{3}$ group to that at ∼1631 cm${}^{-1}$. The normal-mode-based frequencies differ by the same amount as those computed in explicit solvent. However, the comparatively higher frequency of the normal modes assigned to the carbonyl groups indicate effects not contained in the calculations in implicit solvent, such as temperature or, more likely, explicit interactions with the solvent.

### 3.3. Normal Modes of ALAL–Water Clusters

In order to analyze the impact of a hydrogen-bonded water molecule on the individual C=O groups, we performed normal mode calculations of the ALAL peptide hydrogen-bonded to one or more water molecules.

Each of the C=O groups exhibits, as anticipated, lowered stretching frequencies when a water molecule is hydrogen-bonded to it (see Figure S2). The red-shift compared to the frequencies of the unbound ALAL peptide is about 29 cm${}^{-1}$ for all the carbonyl groups, resulting in an about 10 cm${}^{-1}$ higher frequencies than those calculated from the first-principles MD with full explicit solvation.

It is interesting to note that not only the frequency of the normal mode of the carbonyl group that carries the water molecule is affected, but also the other two carbonyl groups show small changes in their stretching frequencies to higher or lower values. With one water molecule hydrogen-bonded at each of the carbonyl groups (three water molecules in total), the red-shifts are slightly different for the three carbonyl groups (25, 27, and 31 cm${}^{-1}$, for the $C_{1}=O_{1}$, $C_{2}=O_{2}$, and $C_{3}=O_{3}$ group, respectively). Note that the water molecules are hydrogen bond donors to the respective carbonyl group and at the same time hydrogen bond acceptors to the neighboring ND groups. Adding one more water molecule at the COO${}^{-}$ group hardly affects the frequencies of the C=O groups.

An extreme is a water cluster in which all polar groups are involved in at least one hydrogen bond (see Figure S2). For such a case, the computed normal modes are 1595, 1577, and 1620 cm${}^{-1}$, for the $C_{1}=O_{1}$, $C_{2}=O_{2}$, and $C_{3}=O_{3}$ group, respectively. That is, no change in frequency is observed for the $C_{3}=O_{3}$ group compared to the unbound peptide. In contrast, the frequencies of the other two carbonyl groups show significant red-shifts. The $C_{2}=O_{2}$ group is involved in more than one hydrogen bond and one might therefore expect a strong red-shift.

Computing the normal modes for the central carbonyl group, $C_{2}=O_{2}$, with one or two hydrogen-bonded water molecules at only this group, confirms the idea of stronger red-shifts by more hydrogen-bonded partners, since one hydrogen bond results in a frequency of 1601 cm${}^{-1}$ whereas two hydrogen bonds lead to a frequency of 1582 cm${}^{-1}$ for the stretching vibration of the $C_{2}=O_{2}$ group, but not quite as much as in the model with hydrogen bonded water molecules at all polar groups.

Such “clean” scenarios are, however, not representative of the full hydration in explicit water. As can be seen from the calculated $C_{2}=O_{2}$ stretching frequencies, different topologies of hydrogen-bonded water molecules (numbers of water molecules and different connections between the polar groups) around the ALAL peptide have different, and sometimes even opposing, effects. Thus, not only to go beyond the harmonic approximation, but also to obtain a more comprehensive picture of the effect of the water solvation on the carbonyl frequencies of the ALAL peptide, it is important to have a closer look at the first-principles MD simulations.

### 3.4. Analysis of the Hydration Shell

**Figure 4:**
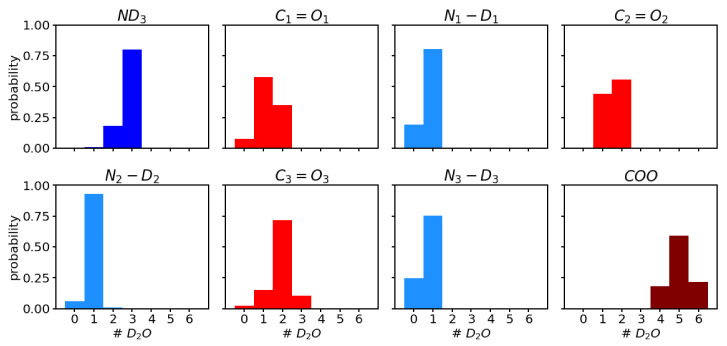
Probability distribution of number of $D_{2}O$ molecules hydrogen-bonded to the polar groups i.e., $ND_{3}$, $C=O^{\prime }s$, $N-D^{\prime }s$ and COO, of the ALAL peptide.

Analysis of the number of water molecules that form a hydrogen bond to the polar groups (see Figure 4) shows a trend of higher probability for more hydrogen-bonded water molecules from $C_{1}=O_{1}$, over $C_{2}=O_{2}$ to $C_{3}=O_{3}$. The average number of hydrogen bonds for the three carbonyl groups $C_{1}=O_{1}$, $C_{2}=O_{2}$, and $C_{3}=O_{3}$, are 1.3 ± 0.6, 1.6 ± 0.5, and 1.9 ± 0.6, respectively. This trend is in agreement with the order of the observed C=O frequencies in the sense that the most red-shifted C=O vibration corresponds to the carbonyl group that has, on average, the highest number of hydrogen-bonded water molecules. In other words, for $C_{3}=O_{3}$, the C=O bond is weakened most often, for $C_{2}=O_{2}$ second, and for $C_{1}=O_{1}$ least, by the presence of a hydrogen bond, and hence the vibrational frequency, which is also computed from an average of all the hydrogen-bonded (or not) scenarios, is more, or less, shifted to lower frequencies.

**Figure 5:**
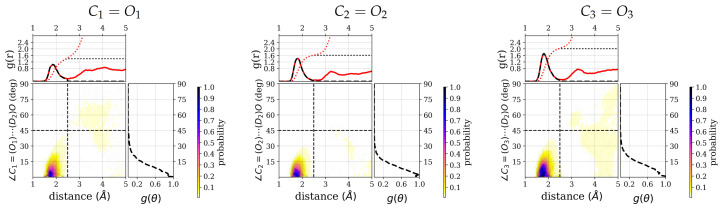
Combined radial distribution functions, g(r), and angular distribution functions, g(θ), of hydrogen-bonded water (D-atoms) around the three carbonyl groups of the ALAL peptide. For the other groups, see Supplementary Figure S3. Each top marginal plot shows g(r) and right marginal plot shows g(θ) for the respective distribution function. A black dashed line is used to show the restriction according to the hydrogen bond criteria. In each g(r) plot, the black and red dotted curves represent the running integration of hydrogen-bondedwater molecules and of all water molecules, respectively.

Closer inspection of the positional distribution of the hydrogen-bonded water molecules in terms of the hydrogen-accpetor distance and the donor–hydrogen–acceptor angles shows a well-defined first solvation region for all three carbonyl groups (see Figure 5) and also for the other polar groups (see Figure S3). The highest density regions are confined to distances of 1.5 to 2.2 Å and angles between 0 and 15 degree deviation from linearity for all polar groups. The integrated number of water molecules that obey at least one hydrogen bond criterion, that is, distance or angle criterion, for the three carbonyl groups are 1.5, 1.6, and 2.0, respectively. Albeit the difference is less pronounced, in particular between the $C_{1}=O_{1}$ and $C_{2}=O_{2}$ group, these numbers follow the same trend as the numbers of water molecules that are within both criteria, i.e., the number of hydrogen-bonded water molecules. Beyond the hydrogen-bonded region, as defined by hydrogen–acceptor distance and hydrogen bond angle, there is still a non-negligible probability for water molecules to be close to, i.e., with a distance below 5Å, and therefore possibly interacting with the carbonyl groups.

For all three carbonyl groups, the number of hydrogen-bonded water molecules fluctuates by about half a molecule. Since this an averaged deviation from the mean, the carbonyl groups actually switch between states in which they have one hydrogen-bound water molecule more or less.

To analyze this further, we have taken a closer look at the water structure around the central carbonyl group, $C_{2}=O_{2}$, whose frequency and number of surrounding/hydrogen-bonded water molecules, is between that of the other two carbonyl groups. Furthermore, this carbonyl group is in a central position, that is least affected by the charged termini, ND${}_{3}^{+}$ and COO${}^{-}$, respectively, and therefore the best representative of a carbonyl group in a longer peptide or protein.

**Figure 6:**
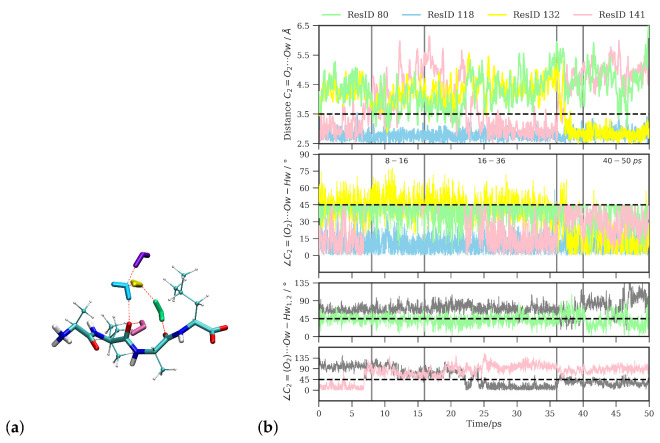
(**a**) Water molecules closest to the central carbonyl group ($C_{2}=O_{2}$). (**b**) Time series of distances and angles between these water molecules and the $C_{2}=O_{2}$ group. The ResID labels are used solely to distinguish and refer to the individual water molecules.

Looking at the time series of water oxygen–carbonyl oxygen distances and wO-H⋯O angles (see Figure 6b) of the water molecules that are closest to the $C_{2}=O_{2}$ group (see (Figure 6a)), one can indeed see changes in the local water structure. One water molecule (labeled as resid 118 for the purpose of distinguishing the individual water molecules) stays close (within 3.5Å) to the carbonyl oxygen atom throughout the simulation time and in an angle within the hydrogen bond criteria most of the simulation time. Between ∼8 and 16 ps, this is the only water molecule that qualifies for a hydrogen bond. Before 8 ps and after 16 ps simulation time, another water molecule (resid 141) is close enough to the $C_{2}=O_{2}$ group and at the correct angle to also be counted as hydrogen-bonded, and yet another water molecule (resid 80) transiently comes close enough to be a candidate for a hydrogen bond. Between 16 and 36 ps simulation time, there is again a rather stable state with two water molecules (resid 118 and 141) in hydrogen-bonded position and orientation to the $C_{2}=O_{2}$ group. At about 36 ps simulation time, the second water molecule (resid 141) leaves again and is replaced by a third water molecule (resid 132) that has been at about 4–4.5 Å distance until then, located in the middle of a three-water bridge to the $C_{3}=O_{3}$ group. As this water molecule moves closer to the $C_{2}=O_{2}$ group, such that it forms a hydrogen bond, the bridge breaks, and is transiently replaced by a two-water bridge. This water molecule stays at the $C_{2}=O_{2}$ group until the end of the simulation (50 ps), rendering this last window again a state with two hydrogenbonded water molecules (see Figure 6). Based on geometric criteria, the average numbers of hydrogen bonds between water and the $C_{2}=O_{2}$ group for these three time windows, i.e., 8–16 ps, 16–36 ps, and 40–50 ps, are 1.0±0.2, 1.5±0.5, and 1.7±0.5, respectively.

**Figure 7:**
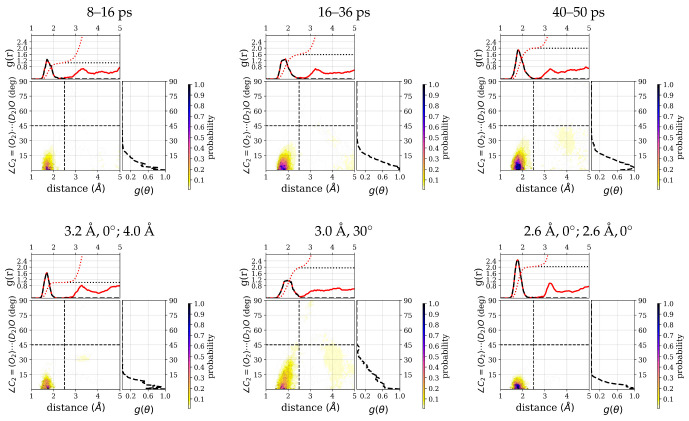
Radial distribution functions, g(r), and angular distribution functions, g(θ), of hydrogen-bonded water (D-atoms) around the $C_{2}=O_{2}$ group of the ALAL peptide, computed for three different time windows: 8–16 ps, 16–36 ps, and 40–50 ps and from constrained simulations (see methods for details). Each top marginal plot shows g(r) and right marginal plot shows g(θ) for the respective distribution function. A black dashed line is used for to show the restriction to hydrogen bond criteria. In each g(r) plot, the black and red dotted curves represent the running integration of hydrogen-bonded water molecules and of all water molecules, respectively.

Correspondingly, the distribution of water molecules around the $C_{2}=O_{2}$ group has a high density in the hydrogen-bonding region (see Figure 7) and some additional low density at a distance around 4 Å that is comparatively higher for the 40–50 ps window than for the other two windows. The integrated number of water molecules in the hydrogen-bonded region for the three time windows are 1.1 (8–16 ps), 1.6 (16–36 ps), and 2.0 (40–50 ps), respectively.

**Figure 8:**
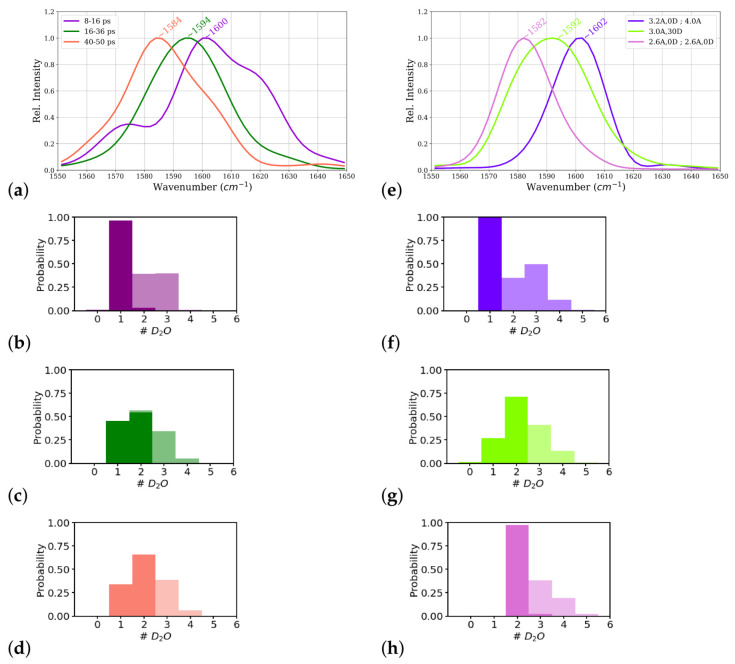
Power spectrum of the central carbonyl group, $C_{2}=O_{2}$, of the ALAL peptide (For the full range power spectra see supplementary Figure S5), computed from (**a**) three time windows of the first-principles simulation: 8–16 ps, 16–36 ps, and 40–50 ps simulation time and (**e**) constrained simulations. Hydrogen bond probabilities as quantified by number of hydrogen-bonded water molecules in the strong (opaque) and moderately (transparent) interacting zone (see Figure 9 for definition), computed from time windows of the unconstrained simulation (**b**) 8–16 ps, (**c**) 16–36 ps, and (**d**) 40–50 ps) and from constrained simulations (see methods for details) (**f**) 3.2Å,0${}^{\circ }$; 4.0Å, (**g**) 3.0Å, 30${}^{\circ }$, and (**h**) 2.6Å, 0${}^{\circ };$ 2.6Å, 0${}^{\circ }$.

Power spectra computed from these time windows of this simulation, corresponding to the one-water, one- and two-water, and two-water situations, indeed show different carbonyl frequencies for these different parts of the trajectory (see Table 1 and Figure 8a). The part that corresponds to a one-water molecule close to the $C_{2}=O_{2}$ group shows a higher frequency (1600 cm${}^{-1}$) than the other two parts (1594 cm${}^{-1}$ and 1584 cm${}^{-1}$, respectively). The frequency computed from the middle part with a mixed state of one and two water molecules close to the $C_{2}=O_{2}$ group shows almost the same frequency as the power spectrum computed from entire trajectory. These data confirm the carbonyl frequency to be a results of the averaged interactions of the water molecules with the $C_{2}=O_{2}$ group.

### 3.5. Interaction Energies

**Figure 9:**
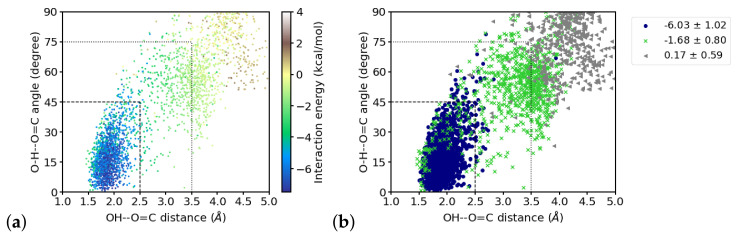
Distribution of interaction energies of the four water molecules closest to the $C_{2}=O_{2}$ group over hydrogen-bond distances and hydrogen bond angles. (**a**) energy values indicated by color, (**b**) interaction energies clustered into “strong” (navy circles), “moderate” (green crosses) and “weak” (grey triangles) interactions. The dashed lines indicate the used hydrogen bond criteria and mark the “strong interaction zone”. The dotted lines mark the “moderate interaction zone”.

The interaction energies between the CONH fragment containing the $C_{2}=O_{2}$ group and individual water molecules vary between almost nothing for the more distant water molecules and ∼6 kcal/mol, which is about the upper limit for the strength of hydrogen bonds [65]. Figure 9 shows the distribution of distances and angles to the $C_{2}=O_{2}$ group of the closest four water molecules and their interaction energies. Within the region that is considered to be hydrogen-bonded, as by hydrogen–acceptor distance and donor–hydrogen–acceptor angle (indicated by dashed lines in Figure 9a), the interaction energies are strongest. Note, however, that toward shorter distances, i.e., at ∼1.6 Å and below, the interaction energies are less favorable. With larger distances and angles, the interaction energy strength generally decreases. One can, however, group these “outer” region also in different zones, based on the (average) interaction energies observed there. Doing this by *k*-means clustering with three clusters, a “strongly interacting zone” can be recognized that coincides with the hydrogen-bonded region (indicated by dashed lines in Figure 9b), a “moderately interacting zone” at larger distances and angles, but below 4.5 Å and 75${}^{\circ }$ (indicated by dotted lines in Figure 9b), and a “weakly interacting zone” (at even larger distances and angles) can be distinguished. The classification into “strong”, “moderate” and “weak” is based on the average interaction energies within the clusters (see Figure 9b). Note that there are also water molecules that geometrically qualify as “strongly hydrogen-bonded” but are members of the “moderately interacting” cluster. Grouping the data into two clusters mainly results in hydrogen-bonded and not hydrogen-bonded water molecules whereas a grouping into four cluster partitions the hydrogen-bonded zone into two groups of “strong” and “very strong” interactions and a separation into “moderately” and “weakly” interacting (see Figure S6), comparable to the results for three clusters. In order to use geometric criteria for a qualitative description of interaction strengths, we decided to use three clusters, i.e., hydrogen-bonded corresponds to “strong”, other close water molecules are classified as “moderately” interacting, and the remaining water molecules are considered as weakly or noninteracting.

Inspecting the interaction energies, and the resulting number of hydrogen-bonded water molecules in the two interacting zones for the time windows of the simulation, we find the closest water molecule (W1) that is hydrogen-bonded throughout the simulation to be interacting with a strength that varies only slightly, i.e., within the error margin, between the three windows (see Table 1). The interaction energies computed for the second closest water molecule (W2), however, differs significantly between the first window (−1.8 ± 1.3 kcal/mol) and the other two windows (−4.6 ± 2.2 and −5.6 ± 1.6 kcal/mol, respectively; see Table 1). This is in agreement with the larger average distance (3.2 ± 0.5Å) of this water molecule in the first window than in the other two (2.5 ± 0.6Å and 2.0 ± 0.2Å, respectively) and also the larger angle (66.1 ± 21.1, 35.5 ± 24.3, and 22.6 ± 15.0 in the first, second, and third window, respectively; see Table 1). Indeed, we find a considerable correlation between the distances, and also the angles, of the second (and third) water molecule and the interaction energies, but only a correlation with the angle for the first water molecule (see Table S1).

As already noted before, in the first window, there is on average only one (strong) hydrogen bond formed between the $C_{2}=O_{2}$ group and a water molecule, W1, whereas in the other two windows, another water molecule, W2, is (strongly) hydrogen bonded for almost the entire last window (40–50 ps) and partially in the second window (16–36 ps). In those frames where W2 is just outside the (strong) hydrogen-bonded zone, it is still close enough to the $C_{2}=O_{2}$ group to interact (probably more than “moderately”) as manifested by the rather strong interaction energy calculated for this water molecule. Within error, this interaction energy is comparable to that computed for the last window, albeit the fluctuation, i.e., the error, is significantly larger.

Relating these interaction energies with the frequencies computed for the three windows, the weak interaction in the first window indeed corresponds to the lowest red-shift (to 1600 cm${}^{-1}$). Regarding the other two windows, the red-shift is largest (to 1584 cm${}^{-1}$) in the last window with the highest average number of strongly hydrogen-bonded water molecules and most favorable interaction energies. Both these values are still close, at least within error, to those of the second window for which an intermediate red-shift (to 1594 cm${}^{-1}$) has been computed. One can therefore argue that it is either the combined effect of two close water molecules interacting less strongly with the $C_{2}=O_{2}$ group in the second than in the last window, or, and probably in addition, the larger fluctuations in the second window, which give rise to the lower red-shift.

The third and fourth water molecules are at the edge of the “moderately” interacting zone, as also manifested by the probability distribution of number of water molecules in the two zones (see Figure 8b–d), for the first, second, and third time window, respectively). These more distant water molecules, moreover, show only weak interactions with the $C_{2}=O_{2}$ group in all three time windows. For all four closest water molecules considered in the calculation of interaction energies, the distance of the closest hydrogen atom (among the hydrogen atoms of all four water molecules) to the $N_{2}-D_{2}$ group, which is also part of the fragment, is large enough (∼4.5Å, see Table 1) that one can consider the calculated interaction energies to be dominantly with the $C_{2}=O_{2}$ group.

### 3.6. Instantaneous Frequencies

**Figure 10:**
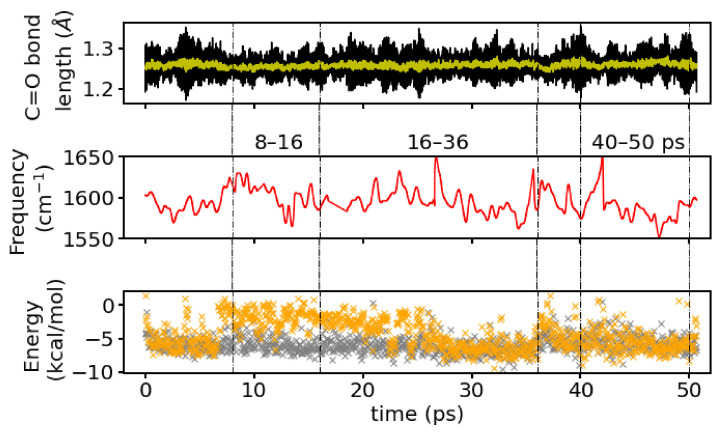
(**Top**): Time series of the $C_{2}=O_{2}$ bond length (black) and its running average (yellow), (**middle**): Instantaneous frequencies from a wavelet analysis, and (**bottom**): interaction energies of the closest (grey) and second closest (orange) water molecule with the $C_{2}=O_{2}$ group. The dashed lines indicate the time windowswhich were analyzed individually.

Figure 10 shows the instantaneous $C_{2}=O_{2}$ frequency (positions of maxima) as calculated from a wavelet analysis. For the full simulation time, the averaged frequencies calculated by the wavelet analysis is 1594 cm${}^{-1}$ which is close to the frequency computed from the Fourier transform of the entire simulation.

The averaged frequency from the wavelet analysis from a first time window of the simulation, 8–16 ps, that corresponds to a situation with one-water hydrogen-bonded to the $C_{2}=O_{2}$ group is 1602 cm${}^{-1}$ and for the two windows that correspond to a mixed and a two-hydrogen-bonds state (16–36 ps and 40–50 ps, respectively) are 1594 cm${}^{-1}$ and 1588 cm${}^{-1}$, respectively. Again, the C=O frequencies for the individual time windows computed by the direct Fourier transform are well reproduced by the wavelets.

The wavelet spectrum contains a number of sudden “jumps” to very low values (<1500 cm${}^{-1}$) that have to be considered artefacts of the transformation not being able to capture some fluctuations in the $C_{2}=O_{2}$ signal properly. These data points have been omitted and smoothed over for clarity in Figure 10. The complete time series of the instantaneous frequencies is shown as supplementary Figure S7, together with the water topology at the $C_{2}=O_{2}$ group, that is the identity of the hydrogen-bonded water molecule(s) and also the hydrogen-bonded connections to other polar groups via hydrogen bonds (water bridges). The artificial “jumps” occur mainly around times, when water molecules exchange positions and/or a water bridge between polar groups forms/breaks or reforms (see Figure S7). A leaving or incoming water molecule distorts the electric field around the $C_{2}=O_{2}$ group and has therefore likely an effect of its bond strength and hence instantaneous frequency. Since there will also be some latency, those changes are not confined to a single frame, but may lead to a response in terms of changed frequency also a few frames after the water positions are rearranged.

Note that the switching between discrete states of formed/broken water bridges suggest the water topology to be more labile than it would appear with an overlapping (instead of binary) definition of hydrogen bonds and thus water bridges. In particular, the last time window at 40–50 ps exhibits frequent changes between existing/non-existing water bridges between the $C_{2}=O_{2}$ group and the $C_{3}=O_{3}$ group or the $COO^{-}$ group of different lengths. The window at 8–16ps, in contrast has a continuous three-water bridge between the $C_{2}=O_{2}$ group and the $C_{3}=O_{3}$ group and transient formation of a bridge to the $COO^{-}$ group, corresponding to a water molecule (marked as resid 80) being close to the $C_{2}=O_{2}$ group or not (see Figure S7).

With one, and the very same water molecule, hydrogen-bonded to the $C_{2}=O_{2}$ group, (instantaneous) changes in the frequency of this group have to be attributed to the other close water molecules. Indeed, the correlation coefficient of the interaction energy of the second closest water molecule (changing between resid 141 to 132) with the instantaneous frequency is 0.4. For the closest water molecules, this correlation is only 0.2 and below 0.1 for the other water molecules. While a correlation of 0.4 is not striking it is certainly not negligible, suggesting that the interaction energies and the instantaneous, and therefore most probably also the averaged, frequencies are related. From comparison of the time series of interaction energies (see Figure 10 middle panel) with the time series of instantaneous frequencies (see Figure 10 bottom panel), one can see a tendency for higher frequencies around times with weaker interactions of the second closest water molecule.

### 3.7. Simulations with Constrained Water Molecules

In order to further probe the effect of water molecules within hydrogen bond distance on the frequency of the $C_{2}=O_{2}$ vibration, we have performed additional simulations, in which one or two water molecules are constrained at different distances (2.6–3.2Å or 4.0Å donor–acceptor distance, see methods for details), such that they are within hydrogen-bonded distance or beyond the cutoff for hydrogen bonds, but still close enough to have an effect.

From the average number of hydrogen bonds, the three constrained simulations correspond to a situation with one (strongly) hydrogen-bonded water molecule (3.2Å, 0${}^{\circ }$; 4Å), two (strongly) hydrogen-bonded water molecules (2.6Å, 0${}^{\circ }$; 2.6Å, 0${}^{\circ }$), and a mixed situation (3.0Å, 30${}^{\circ }$), comparable to the three windows in the unconstrained simulation (see Table 1). The integrated number of hydrogen-bonded water molecules of 1.0, 2.0, and 2.0 for the 3.2Å, 0${}^{\circ }$; 4Å constraints, 3.0Å, 30${}^{\circ }$ constraints, and 2.6Å, 0${}^{\circ }$; 2.6Å, 0${}^{\circ }$ constraints, follow essentially the same trend (see Figure 7).

The carbonyl frequencies computed for the constrained simulations are, $∼1582,∼1592,\;\mathrm{and}\;∼1602\;\mathrm{cm}{}^{-1}$ for the two-water, mixed, and one-water molecule scenarios, respectively (see also Table 1 and Figure 8e), similar to those observed in the time windows of the corresponding states. The interaction energies between close water molecules and the $C_{2}=O_{2}$ group are similar to those computed for the time windows with comparable scenarios: the closest water molecule interacts strongly in all the constrained simulations, and the second water molecule, again, interacts strongest in the two-water case and weakest in the one-water case. In the latter scenario, this second water molecule is again on the border between the “strong” and “moderately” interacting zone as far as distances and angles are concerned, but its interaction energies rather classifies it as a member of the “moderately interacting” group. As also observed in the unconstrained simulation, the third and fourth water molecule interact only weakly, with a little stronger interactions of the third water molecule in the two-water or mixed scenarios. The distance to the $N_{2}-D_{2}$ group is for all four water molecules large enough to again consider the interaction energies to be dominated by the interaction with the $C_{2}=O_{2}$ group. There is a probability of ∼0.3 or more to find a third water molecule in the “moderately interacting zone” in all the cases, that is one-water, two-water or mixed scenarios, modeled in the time windows of the unconstrained simulation (see Figure 8b–d) and the different constrained simulations (see Figure 8f–h). Hence, it is not possible to tell whether the presence of this third water molecule has a direct impact on the $C_{2}=O_{2}$ frequency or not. It is however, conceivable that this water molecule contributes indirectly by connections to the strongly hydrogen-bonded water molecule(s) and maintaining the water topology, e.g., water bridges to other polar groups, in the hydration shell of the $C_{2}=O_{2}$ group.

## 4. Discussion

Our analyses show that the interactions between water molecules and the $C_{2}=O_{2}$ group clearly have an effect on the stretching frequency of this carbonyl group. From the normal mode analysis of isolated ALAL–water clusters, it becomes apparent that more hydrogen-bonded water molecules lead to a more pronounced red-shift, but also that in water clusters with several water molecules bound to the different polar groups of the peptide, thought to be more representative of an actually solvated peptide, the hydrogen-bonded situation is too complicated to allow for a simple prediction of (possible) red-shifts. MD simulations in explicit water are therefore a great way to sample different, more or less complex, water topologies in the hydration shell of the peptide. The agreement of the trends in red-shift of the carbonyl frequencies and average number of hydrogen-bonded water molecules observed in our first-principles MD simulation of the ALAL peptide in water corresponds to the, perhaps idealized, trend in red-shift by hydrogen-bonding one or two water molecules to the $C_{2}=O_{2}$ group. In fact, the frequencies computed by the two approaches, i.e., MD and normal modes, are in a striking agreement: (on average) one hydrogen-bonded water molecule at the $C_{2}=O_{2}$ group leads to a frequency of ∼1600 cm${}^{-1}$ and (on average) two water molecules hydrogen-bonded to the $C_{2}=O_{2}$ group result in a frequency of ∼1582 cm${}^{-1}$. While one can argue that this shows how well the implicit solvent approach models the average effect of the explicit solvent, being aware of the other approximations in the normal mode analyses, i.e., harmonic model and zero temperature, we consider the almost exact match of the frequencies rather as a coincidence, but appreciate the similarities. They give us some confidence that one-water and two-water states can, to some extent, be mimicked by the respective water clusters.

Our 100 ps long first-principles MD simulation is sufficient to sample one-water and two-water states, with full hydration, and also something we call a mixed state, that is a period in which the hydrogen-bonded states change between one and two. Since these states (luckily) prevailed long enough in our present simulation, we were able to obtain spectra from the different states that still contain a dynamic average of the system. The simulations with water molecules constrained such that one-water, two-water and mixed states prevail by construction, lead to similar results in the carbonyl frequencies, confirming the averages obtained from the (shorter) time windows to be sufficient.

The calculation of interaction energies between the closest water molecules and the $C_{2}=O_{2}$ group (as the CONH fragment) clearly demonstrate the (weakening) effect of strong interactions with the water molecules on the $C_{2}=O_{2}$ bond by the resulting red-shifts of the vibration frequency. Since throughout all the MD simulations one water molecule is strongly interacting with the $C_{2}=O_{2}$ group, differences in the red-shifts have to be attributed to other water molecules, and it turns out that the interaction strength of a second water molecule, and fluctuations therein, can indeed explain this effect. A linear correlation coefficient between the fluctuations of the interaction energies of this second water molecule and the instantaneous frequencies, computed by a wavelet analysis, of 0.4 confirms the relation between water - $C_{2}=O_{2}$ interaction, but also reveals that there are other effects, and/or higher-order correlations, that need to be considered. Such other effects are found, at least qualitatively, as changes in the water topology, i.e., water bridges connecting the $C_{2}=O_{2}$ group and the other polar groups, which occur around times when also the instantaneous frequencies exhibit large jumps. This is again indicative of the higher order hydration shells also affecting the carbonyl frequencies and this has at least to be averaged out, to render the somewhat simple minded one-water or two-water states to be sufficient descriptors.

Relating the computed interaction energies with the water-hydrogen–carbonyl-oxygen distances and water oxygen-hydrogen–carbonyl oxygen angles, that is hydrogen–acceptor distances and donor–hydrogen–acceptor angles that are typically used to geometrically define hydrogen bonds, we find a strong correlation of the interaction energies with both, the distances and the angles for the second water molecule. By clustering the interaction energies we could identify a “strongly interacting zone” that coincides with the geometric criteria for hydrogen bonds, often used in the MD community: 2.5 Å of maximal hydrogen–accpetor distance and a donor–hydrogen–acceptor angle that deviates by at most 45${}^{\circ }$ from linearity. (When analyzing MD simulations with classical force fields, the distance criterion is often taken as 3.5 Å of maximal donor–acceptor distance, but with ∼1Å as the typical donor–hydrogen distance, these two distance criteria can be considered equivalent.) This is also the hydrogen bond criterion used for a geometrical definition of a hydrogen bond in the present study. The distribution of interaction energies over the hydrogen bond distance/hydrogen bond angle space, however, reveals also that the interactions in the “strongly interacting”, hydrogen-bonded zone are not necessarily strong since in some frames water molecules with a correct hydrogen-bonded position interact only moderately. In turn, also with water molecules positions outside the hydrogen-bonded region, strong interactions with the $C_{2}=O_{2}$ group are occasionally computed. We therefore stress that, though the geometric criteria has been confirmed by the averaged interaction energies in the hydrogen-bonded zone, this geometric definition is useful for looking at probabilities for hydrogen bonds, taken from averaging over many water positions. If used in this manner, a simple geometric criterion is indeed a fast and representative metric to describe the hydrogen-bonded state of a system, or at least a carbonyl group surrounded by water molecules.

The different hydrogen-bonded scenarios of the $C_{2}=O_{2}$ group observed in the time windows and constructed by constraints, representing the averaged interactions between the water molecules and the $C_{2}=O_{2}$ group, can thus be used to qualitatively explain the observed red-shifts in the vibrational frequency. We are furthermore confident that the different probabilities of the three carbonyl groups in the ALAL-peptide to form hydrogen bonds with the solvent can also be used to explain the observed differences in their individual vibrational frequencies. The averaged number of hydrogen bonds simply has to be considered not the cause of a red-shift but rather a marker for a hydration situation with water-carbonyl interactions that leads to such a shift.

The definition of a hydrogen bond is something scientists argue about since at least Pauling and the one found in a IUPAC technical report (“The hydrogen bond is an attractive interaction between a hydrogen atom from a molecule or a molecular fragment X–H in which X is more electronegative than H, and an atom or a group of atoms in the same or a different molecule, in which there is evidence of bond formation.” [66]) may be precise but not directly helpful. Even in the very publication, several ways to provide “evidence of bond formation” are presented, and the “attractive interaction” or “nature of physical forces” are among them.

Different hydrogen bond criteria have been evaluated in e.g., [67,68,69,70,71], all having their different merits. The most reassuring statement is probably, “The fact that different choices for the relevant geometric variables and a quite different electronic structure approach all lead to quite similar results for both the statics and dynamics of H-bond number fluctuations does perhaps suggest that these ways [i.e., gemeometric definitions] of considering H bonds in the liquid can be insightful.” [69]. Another consensus is that the relation between water position, i.e., distance and angles, with respect to the interaction partner, is related to the interaction strength as also found in the present study. The difficulty is rather where, not whether or not, to put the cutoffs, be it on the energy scale or geometrically. Our compromise by clustering the interaction energies and determine the (“strongly interacting”) hydrogen-bonded zone from the distance/angle distribution of the cluster members reduces some of the arbitrariness but is of course unnecessary if one chooses to work with interaction energies directly.

One also needs to keep in mind that the fragmentation approach used to calculate the interaction energies introduces errors (due to the capping hydrogen atoms) that can in principle be different for the various frames. Correction methods such as scaling the capping hydrogen atoms [72] or using embedded charges as done in another fragmentation method [73] exist. Having a decent number of data points, though, we are confident that such errors are averaged out.

In recent works, both the distance to a hydrogen-bonded partner as well as the strength of a hydrogen bond (and the amount of charge transfer) have been found to correlate with the OH stretch frequency in liquid methanol [74] or water [26,75] or the ND stretch frequency in NMA [76]. These correlations have been determined by comparing the instantaneous frequency of the stretching vibrations, as computed by a wavelet analysis of first-principles MD simulations, with the hydrogen bond distances [21,75,76] or the hydrogen bond strength [26]. In our work, we relate solute–solvent interactions with solvent vibrations, and therefore have significantly fewer data points than available for solvent–solvent interactions and corresponding frequencies. This may be one reason why the correlation between interaction energies and instantaneous frequencies of the $C_{2}=O_{2}$ vibration are less pronounced than in other works in the literature. Still, the approach has proved useful to identify the relations.

Even without a detailed analysis of the instantaneous frequencies, computing vibrational spectra of solvated peptides by first-principles MD simulations provides significant insight into the dynamics of the molecule in water and the effect of the solvent on the (calculated) vibrational properties [9,10,11,12]. The carbonyl frequencies computed in this work are in the same range as those computed for other peptides of similar size [11,77,78]. For example, the calculated frequency of the alanine dipeptide is 1605 cm${}^{-1}$ and the experimental value is 1635 cm${}^{-1}$ [79]. Our present results are in particular comparable to those of to the Ala-Leu peptide, with measured carbonyl vibration at 1660 cm${}^{-1}$ and a calculated frequency at ∼1600 cm${}^{-1}$ [14]. This is the same frequency that we find in this work for the central carbonyl group in a one-water state, in agreement with the one hydrogen bond (on average) observed for the carbonyl group in Ala-Leu [14], confirming again the interplay of hydrogen bonds and vibrational frequencies.

## 5. Conclusions

The present analysis of the hydration shell around the ALAL peptide and, in particular, the central $C_{2}=O_{2}$, carbonyl group, afforded us to closer inspect the factors that influence the vibrational frequency, and thus the spectroscopic signature, in the amide I region. Differences in the frequencies of the three individual carbonyl groups can be attributed to their interactions with the surrounding water. The probabilities of the groups to form hydrogen bonds with water is in agreement with the observed shifts in the computed stretching frequencies.

States in which the central carbonyl group has one or two hydrogen-bonded water molecules, or a mixture thereof, (either observed over time windows of an unconstrained simulation or constructed by constraints) exhibit a clear trend of the red-shift of the $C_{2}=O_{2}$ vibrational frequencies with the averaged number of hydrogen-bonded water molecules. The amount by which the frequencies are lowered is reflected in the strengths of the interaction energies between the closest water molecules and the peptide fragment containing the $C_{2}=O_{2}$ group. Since one water molecule is strongly interacting throughout the simulations, it is in particular the second water molecule that is decisive for finding one or two strongly interacting, and also hydrogen-bonded, water molecules. It is this second interaction that determines the amount of the (additional) red-shift.

The geometric definition of a hydrogen bond by distance and angle criteria, typically used in the MD community and also in this work, is justified by the distribution of interaction energies between water molecules and the $C_{2}=O_{2}$ group of the ALAL peptide over water–carbonyl distances and angles. Since, however, strong interactions are also observed for water molecules that are outside the geometric cutoffs and, likewise, weaker interactions for water molecules within the criteria for hydrogen bonds, the geometric definition holds only on average. Still, when used together with ensemble averages or dynamical averages, the average number of (geometrically defined) hydrogen bonds can serve as a qualitative representative of stronger or weaker interactions which, in turn, more strongly or more weakly, impact the strength of, e.g., a carbonyl bond, and thus its vibrational frequency. With first-principles MD simulations, these averages can be computed, providing simultaneous insight into the dynamical interaction of water with the peptide and the vibrational dynamics of the individual groups involved.

## Figures and Tables

**Table 1:** Frequencies of the stretching vibration of the second carbonyl group, $C_{2}=O_{2}$, in the ALAL peptide in solution, corresponding to the highest peak of the respective power spectrum (see also Figure 8). Interaction energies of the four closest water molecules with the $C_{2}=O_{2}$ group, distances and angles of the closest hydrogen atom to the $C_{2}=O_{2}$ group, and distances of the closest hydrogen atom of the closest water molecules to the $N_{2}-D_{2}$ group. #$D_{2}O$ denotes the number of water molecules within the “strong”, i.e., hydrogen-bonded, and “moderately” interacting zone (see text for definitions). The four water molcules are ranked and (re)labeled W1, W2, W3, or W4 by their oxygen atom distances to oxygen atom of the $C_{2}=O_{2}$ group at each frame such that, e.g., W2 can refer to different individual water molecules.

	Unconstrained				Constrained		
	Full (0–50 ps)	8–16 ps	16–36 ps	40–50 ps	3.2 Å, 0${}^{\circ }$; 4.0 Å	3.0 Å, 30${}^{\circ }$	2.6 Å, 0${}^{\circ }$; 2.6 Å, 0${}^{\circ }$
Frequency (cm${}^{-1}$)	1592	1600	1594	1584	1602	1592	1582
Energy (kcal·mol${}^{-1}$)							
W1	−5.9 ± 1.2	−5.8 ± 1.1	−6.2 ± 1.2	−5.5 ± 1.3	−6.5 ± 1.3	−5.2 ± 1.0	−5.9 ± 1.0
W2	−4.3 ± 2.3	−1.8 ± 1.3	−4.6 ± 2.2	−5.6 ± 1.6	−1.7 ± 0.8	−5.1 ± 1.0	−6.1 ± 1.0
W3	−0.7 ± 1.0	−1.0 ± 0.8	−0.7 ± 0.9	−1.0 ± 1.2	−1.2 ± 0.7	−0.5 ± 1.6	−1.1 ± 1.2
W4	−0.6 ± 1.1	−0.7 ± 1.2	−0.6 ± 1.3	−0.6 ± 0.8	−0.8 ± 0.8	−0.8 ± 1.2	−0.5 ± 1.0
#$D_{2}O$ (strong)	1.6 ± 0.5	1.0 ± 0.2	1.5 ± 0.5	1.7 ± 0.5	1.0 ± 0.1	1.7 ± 0.5	2.0 ± 0.2
#$D_{2}O$ (moderate)	0.9 ± 0.8	1.3 ± 0.8	0.9 ± 0.8	0.9 ± 0.8	1.9 ± 0.9	1.0 ± 0.8	0.9 ± 0.8
Distance (Å) CO⋯H-Ow							
W1	1.8 ± 0.2	1.8 ± 0.2	1.8 ± 0.2	1.8 ± 0.1	1.7 ± 0.1	1.9 ± 0.2	1.7 ± 0.1
W2	2.5 ± 0.6	3.2 ± 0.5	2.5 ± 0.6	2.0 ± 0.2	3.2 ± 0.3	2.2 ± 0.2	1.9 ± 0.1
W3	3.7 ± 0.5	3.6 ± 0.4	3.7 ± 0.4	3.5 ± 0.5	3.5 ± 0.4	3.6 ± 0.6	3.4 ± 0.5
W4	4.1 ± 0.5	4.1 ± 0.6	4.1 ± 0.6	4.0 ± 0.4	3.9 ± 0.5	3.8 ± 0.5	3.8 ± 0.6
Distance (Å) ND⋯Ow	4.5 ± 0.7	4.5 ± 0.6	4.3 ± 0.8	4.7 ± 0.7	4.7 ± 0.9	4.5 ± 0.8	4.7 ± 0.6
Angle (${}^{\circ }$) CO⋯H-Ow							
W1	17.3 ± 10.1	17.2 ± 9.5	17.2 ± 10.1	19.1 ± 10.6	13.6 ± 7.5	27.8 ± 14.1	12.4 ± 5.2
W2	38.5 ± 27.2	66.1 ± 21.1	35.5 ± 24.3	22.6 ± 15.0	53.7 ± 15.2	30.9 ± 15.6	8.4 ± 4.9
W3	118.8 ± 25.8	125.7 ± 17.8	124.4 ± 20.3	121.7 ± 23.0	57.6 ± 19.9	82.5 ± 28.9	73.3 ± 23.2
W4	78.1 ± 29.0	81.7 ± 26.6	75.9 ± 30.2	77.4 ± 29.1	63.7 ± 28.1	70.2 ± 25.6	80.7 ± 28.0

## Data Availability

Data are available from the authors by request.

## Supplementary Materials

The following are available online, Figure S1: Probability distribution of the side chain torsion angles. Figure S2: Normal mode analysis of different ALAL-water clusters, Figures S3–S4: Combined radial and angular distribution functions, Figure S5: Full range power spectra, Figure S6: Results of *k*-means clustering, Figure S7: Time series of the instantaneous frequencies and water topologies, Table S1: Correlation between interaction energies and $C_{2}=O_{2}\cdots H-Ow$ distances or $C_{2}=O_{2}\cdots H-Ow$ angles.

## Abbreviations

The following abbreviations are used in this manuscript:

ALAL	Ala-Leu-Ala-Leu
NMA	N-methyl amide
MD	Molecular dynamics
IR	Infrared
GPW	Gaussian and plane waves
GTH	Geodecker–Teter–Hutter
DZVP	double zeta valence plus
PCM	polarisable continuum model
DFT	density functional theory
