# Supplementary material for: Effect of the Hydration Shell on the Carbonyl Vibration in the Ala-Leu-Ala-Leu Peptide
Source: Molecules. 2021 Apr 8;26(8):2148. doi: 10.3390/molecules26082148 (PMC8068333; doi:10.3390/molecules26082148)
Supplement: Supplementary file 1 [file molecules-26-02148-s001.pdf]

Supplementary material: Effect of the water dynamics on the carbonyl vibration in the Ale-Leu-Ala-Leu peptide

Irtaza Hassan[1], Federica Ferraro[2], and Petra Imhof[1,2]

6th April 2021

[1] Department of Physics
Freie Universität Berlin
Arnimallee 14
14195 Berlin
Germany

[2] Computer Chemistry Center
Friedrich-Alexander University (FAU) Erlangen-Nürnberg
Nägelsbachstrasse 25
91052 Erlangen
Germany

petra.imhof@fau.de

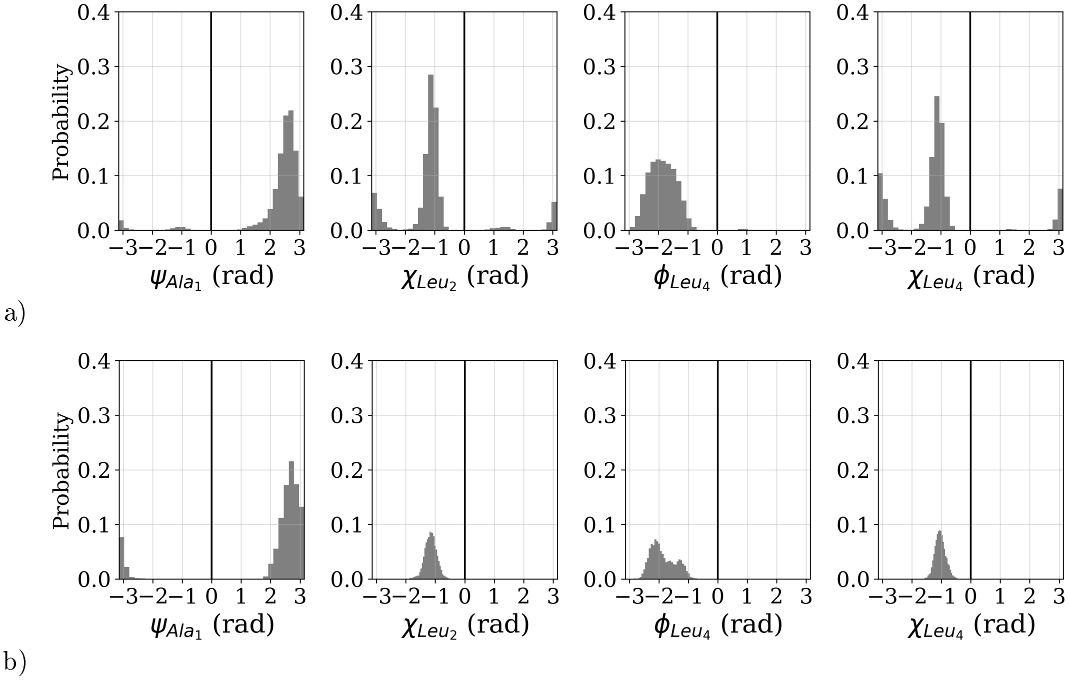

Figure S1: Probability distribution of the χ_1 side chain torsion angles of the Leu residues, χ_{Leu2} and χ_{Leu4} , and the first and last backbone torsion angles, ψ_{Ala1} and ϕ_{Leu4}, computed from a) the classical and b) the first-principles MD simulation of the ALAL peptide in water.

unbound

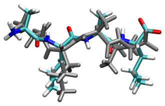

$C_1 = O_1$: 1645
$C_2 = O_2$: 1631
$C_3 = O_3$: 1619
RMSD: 2.15

one hydrogen bond
$C_1 = O_1$

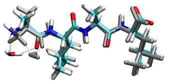

$C_1 = O_1$: 1617
$C_2 = O_2$: 1639
$C_3 = O_3$: 1623
RMSD: 0.83

one hydrogen bond
$C_2 = O_2$

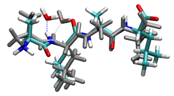

$C_1 = O_1$: 1636
$C_2 = O_2$: 1601
$C_3 = O_3$: 1627
RMSD: 1.34

one hydrogen bond
$C_3 = O_3$

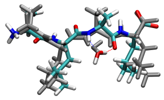

$C_1 = O_1$: 1645
$C_2 = O_2$: 1628
$C_3 = O_3$: 1591
RMSD:.57

C=O groups solvated

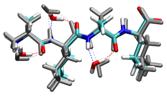

$C_1 = O_1$: 1620
$C_2 = O_2$: 1604
$C_3 = O_3$: 1588
RMSD: 0.60

C=O groups and COO^- group

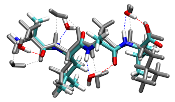

solvated
$C_1 = O_1$: 1622
$C_2 = O_2$: 1604
$C_3 = O_3$: 1588
RMSD: 1.16

two hydrogen bonds
$C_2 = O_2$

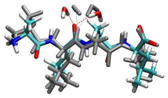

$C_2 = O_2$: 1582

RMSD: 1.05

all polar groups

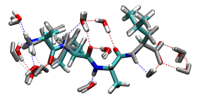

solvated
$C_1 = O_1$: 1595
$C_2 = O_2$: 1577
$C_3 = O_3$: 1620
RMSD: 0.24

0-0-2

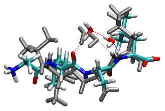

$C_2 = O_2$: 1604
RMSD: 1.48

0-3-0

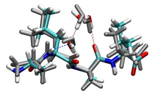

$C_2 = O_2$: 1632
RMSD: 0.58

0-2-2

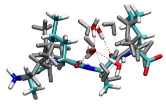

$C_2 = O_2$: 1591
RMSD: 1.83

0-3-3

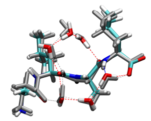

$C_2 = O_2$: 1581
RMSD: 0.39

2-0-0

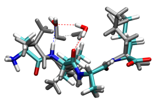

$C_2 = O_2$: 1613
RMSD: 1.63

2-3-0

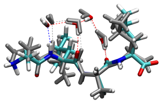

$C_2 = O_2$: 1611
RMSD: 0.94

0-3-2

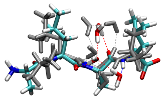

$C_2 = O_2$: 1592
RMSD: 1.83

2-3-3

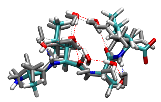

$C_2 = O_2$: 1596
RMSD: 0.86

3-0-0

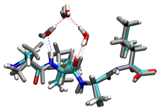

$C_2 = O_2$: 1615
RMSD: 0.60

3-3-0

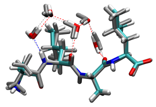

$C_2 = O_2$: 1609
RMSD: 0.30

4-3-0

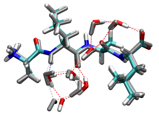

$C_2 = O_2$: 1624
RMSD: 0.29

3-3-3

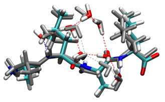

$C_2 = O_2$: 1612
RMSD: 0.57

Figure S2: Frequencies (cm^{-1}) of the carbonyl stretching vibrations, computed by normal mode analysis of different ALAL-water clusters. The hydrogen bonding topology is described or indicated by labels x-y-z for connections between $C_2 = O_2 \cdots N_1 - D_1$ with x water molecules, $C_2 = O_2 \cdots C_3 = O_3$ with y water molecules and $C_2 = O_2 \cdots N_3 - D_3$ with z water molecules, respectively. RMSD is the root mean square deviation (Å) between the initial snapshot (grey) and the optimised (coloured) structure.

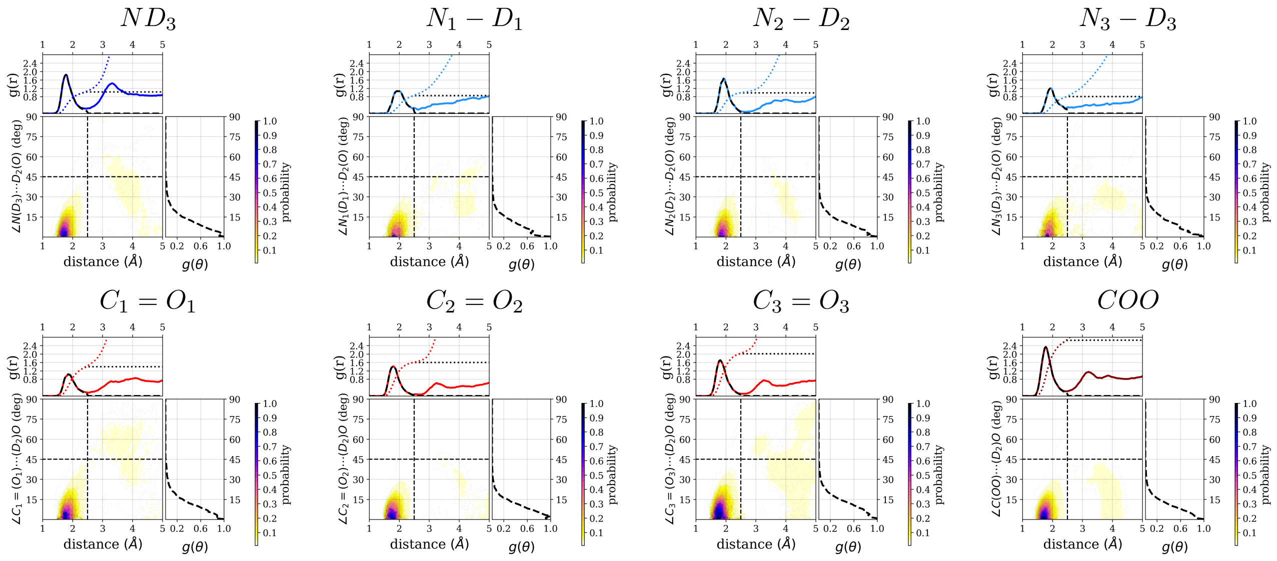

Figure S3: Combined radial distribution functions, $g(r)$, and angular distribution functions, $g(\theta)$, of hydrogen-bonded water (D-atoms) around the polar groups of the ALAL peptide. Each top marginal plot shows $g(r)$ and right marginal plot shows $g(\theta)$ for the respective distribution function. Black dashed line-style is used for to show the restriction to hydrogen bond criteria. In each $g(r)$ plot, the black and red dotted curves represent the running integration of hydrogen-bonded water molecules and of all water molecules, respectively.

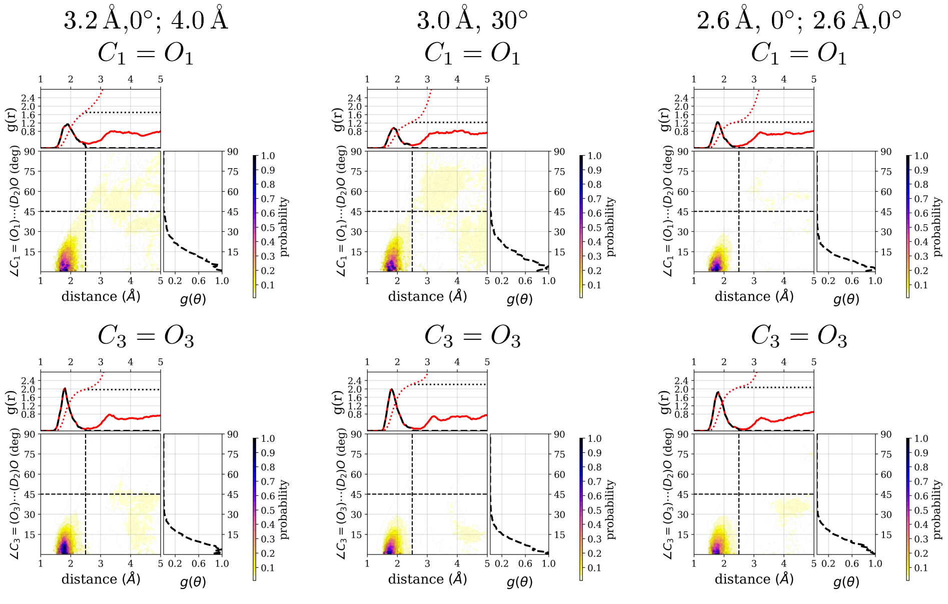

Figure S4: Combined radial distribution functions, $g(r)$, and angular distribution functions, $g(\theta)$, of hydrogen-bonded water (D-atoms) around the $C_1 = O_1$ (top) and $C_3 = O_3$ (bottom) group of the ALAL peptide, computed for the constrained simulations 3.2 Å,0°; 4.0 Å, 3.0 Å, 30°, and 2.6 Å, 0°; 2.6 Å,0°. Each top marginal plot shows $g(r)$ and right marginal plot shows $g(\theta)$ for the respective distribution function. Black dashed line-style is used for to show the restriction to hydrogen bond criteria. In each $g(r)$ plot, the black and red dotted curves represent the running integration of hydrogen-bonded water molecules and of all water molecules, respectively.

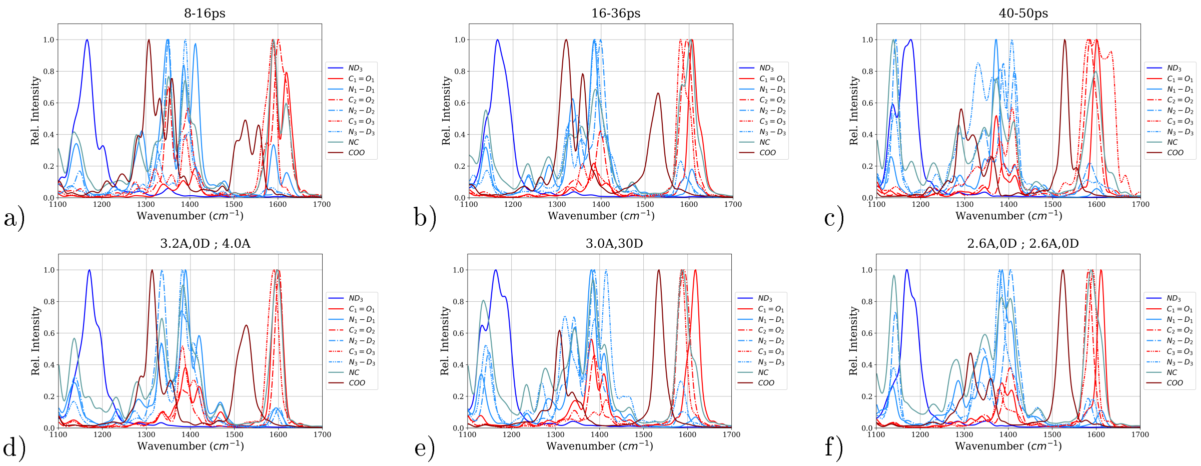

Figure S5: Power spectra computed from first principles calculations of the ALAL peptide in deuterated water from windows a) 8-16 ps, b) 16–36 ps, and c) 40–50 ps of unrestrained simulation, as well as from simulations with restraints (see methods for details) d) 3.2 Å, 0°; 4.0 Å, e) 3.0 Å, 30°, and f) 2.6 Å, 0°; 2.6 Å, 0°.

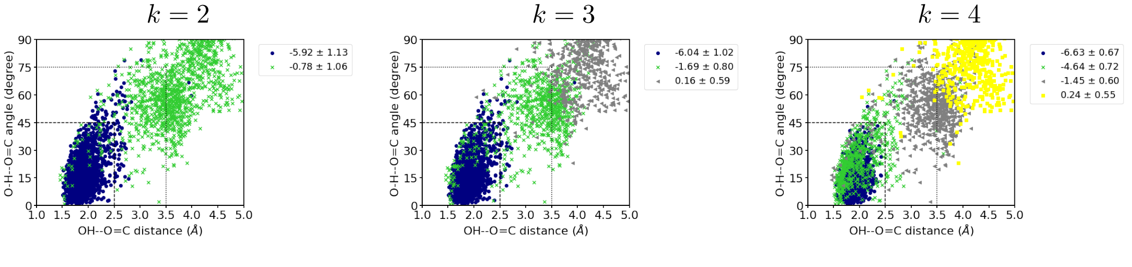

Figure S6: Results of k-means clustering the interaction energy values, computed from the snapshots of an unconstrained simulation of the ALAL peptide in water.

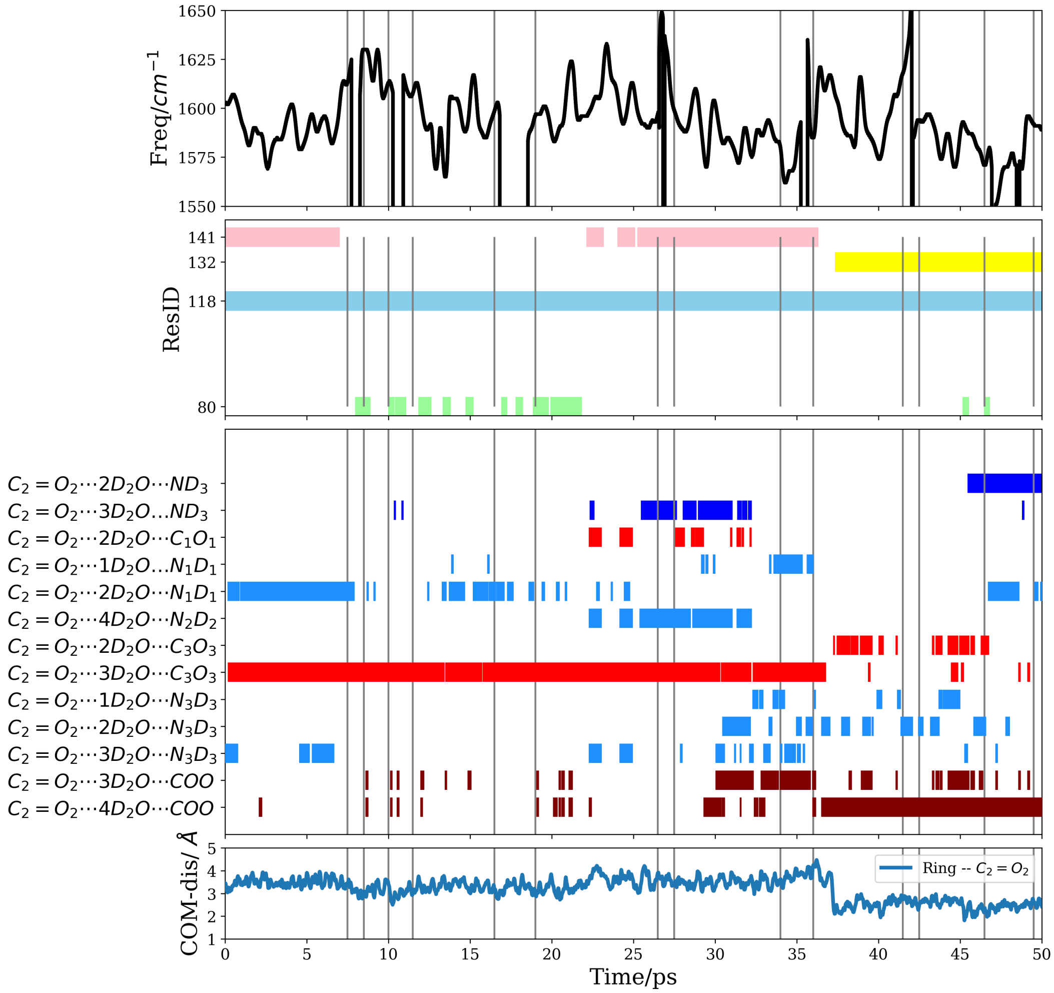

Figure S7: Time series of the instantaneous frequencies from a wavelet analysis, individual water molecules hydrogen-bonded to the $C_2 = O_2$ group (see also Figure 6 in the main text), water bridges between the $C_2 = O_2$ group and the other polar groups, and distance of the centre of mass of a three-water ring (see Figure 6a) in the main text), connecting the $C_2 = O_2$ and $C_3 = O_3$ group to the centre of mass of the $C_2 = O_2$ group.

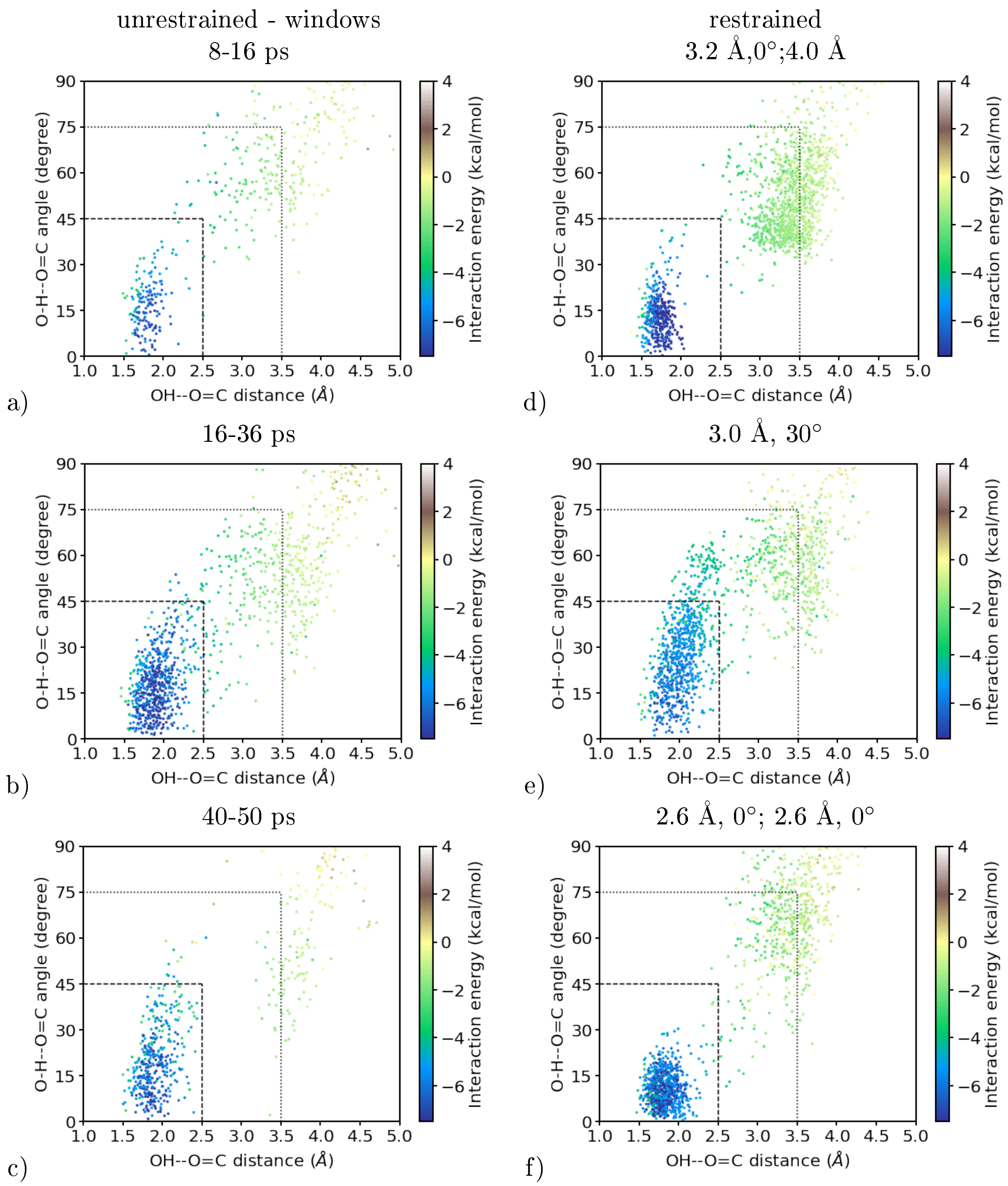

Figure S8: Interaction energy distributions for the four water molecules closest to the $C_2 = O_2$ group from windows if an unrestrained simulation a) 8–16 ps, b) 16–36 ps, and c) 40–50 ps, as well as simulations with restraints (see methods for details) d) 3.2 Å, 0°; 4.0 Å, e) 3.0 Å, 30°, and f) 2.6 Å, 0°; 2.6 Å, 0°. Note that the number of points are different due to the different simulation or window lengths.

Table S1: Correlation between interaction energies and $C_2 = O_2 \cdots H - Ow$ distances or $C_2 = O_2 \cdots H - Ow$ angles.

Restraint	Energy-Distance Correlation W1	W2	W3	W4	Energy-Angle Correlation W1	W2	W3	W4
None (full)	-0.09	0.89	0.80	0.40	0.43	0.83	-0.78	0.47
None (8–16 ps)	-0.22	0.86	0.80	0.30	0.49	0.67	-0.64	0.32
None (16–36 ps)	-0.10	0.91	0.78	0.24	0.38	0.77	-0.69	0.31
None (40–50 ps)	0.03	0.65	0.70	0.74	0.51	0.75	-0.72	0.76
3.0 Å, 30°	0.14	0.76	0.78	0.67	0.49	0.79	0.84	0.70
3.2 Å, 0°; 4.0 Å	-0.56	0.73	0.79	0.75	0.38	0.46	0.66	0.75
2.6 Å, 0°; 2.6 Å, 0°	-0.21	0.27	0.69	0.72	0.01	-0.01	0.65	0.67
